# Does clinical research account for diversity in deploying digital health technologies?

**DOI:** 10.1038/s41746-023-00928-2

**Published:** 2023-10-10

**Authors:** Nathan A. Coss, J. Max Gaitán, Catherine P. Adans-Dester, Jessica Carruthers, Manuel Fanarjian, Caprice Sassano, Solmaz P. Manuel, Eric Perakslis

**Affiliations:** 1 HumanFirst, Inc., San Francisco, CA USA; 2 Department of Anesthesia and Perioperative Care, University of California, San Francisco, San Francisco, CA USA; 3 Duke Clinical Research Institute, Duke University, Durham, NC USA

**Keywords:** Databases, Clinical trial design, Outcomes research

## Abstract

Digital health technologies (DHTs) should expand access to clinical research to represent the social determinants of health (SDoH) across the population. The frequency of reporting participant SDoH data in clinical publications is low and is not known for studies that utilize DHTs. We evaluated representation of 11 SDoH domains in 126 DHT-enabled clinical research publications and proposed a framework under which these domains could be captured and subsequently reported in future studies. Sex, Race, and Education were most frequently reported (in 94.4%, 27.8%, and 20.6% of publications, respectively). The remaining 8 domains were reported in fewer than 10% of publications. Medical codes were identified that map to each of the proposed SDoH domains and the resulting resource is suggested to highlight that existing infrastructure could be used to capture SDoH data. An opportunity exists to increase reporting on the representation of SDoH among participants to encourage equitable and inclusive research progress through DHT-enabled clinical studies.

## Introduction

Clinical research ought to serve all humans, and digital health technologies (DHTs) ought to empower that aim. Clinical research is the means by which life-enhancing and life-saving treatments are brought to the public. In practice, however, many groups have been underrepresented in research, limiting the generalizability of medical breakthroughs^1^. A key intent of DHTs is to broaden access to clinical research by enabling remote monitoring and diminishing patient burden. A number of empirical^2,3^ and review^4–9^ publications demonstrate the potential for DHTs to enhance equity in clinical research and care, though others highlight potential pitfalls^10,11^, including bias and ethical challenges in the use of artificial intelligence and possible amplification of existing inequities in access and digital literacy. Despite this existing work, there are scant data indicating whether clinical research utilizing DHTs enhances participation from underrepresented groups. The scarcity of such information results in part from infrequent and inconsistent reporting on social determinants of health (SDoH)^12^—conditions in which people are born, live, work, learn, and play that impact health and quality of life. Inclusion and reporting of groups negatively impacted by SDoH in clinical research would stand to bridge the gaps that limit generalization of diagnostic or therapeutic modalities.

DHTs comprise a range of physical and software-based tools including wearable sensors, artificial intelligence and machine learning algorithms, and digital therapeutics that enable decentralized ambulatory or at-home health monitoring^13^. DHTs ostensibly improve access to clinical research by reducing the need for burdensome clinical visits imposed by traditional monitoring during clinical trials^14^. For example, cardiac arrhythmias may now be monitored continuously and remotely^15^. Ideally, DHTs also improve the quality and granularity of data to enhance insights into disease treatment.

Clinical trial cohorts ought to represent a spectrum of SDoH in part because SDoH directly impact health outcomes. In *Healthy People 2030*, the United States Department of Health and Human Services has prioritized reducing health disparities linked to SDoH as one of its key initiatives^16^. Furthermore, the *Healthy People 2030* initiative organizes SDoH into domains^16^. This study expanded on this organization and classified each unique SDoH identity within a single SDoH domain as a domain-group. Unfortunately, the representation of SDoH domains among study cohorts may be infrequently reported^17^, which limits confidence as to whether their results are generalizable to the broader population. Information as to the representativeness of DHT-enabled clinical research is particularly important given the intent to use them to enhance trial accessibility.

Advances in medical technology and care resulting from clinical research do not always benefit the population equitably, in part because underrepresented groups have not been able to contribute to development programs. Disparities in cancer outcomes persist among socioeconomically disadvantaged groups^18^ and differences in telemedicine utilization occurred during the COVID-19 pandemic on the basis of gender, race, education, and other demographic characteristics^19^. Significant disparities in participation and device wear time have been demonstrated on the basis of race and income in research involving DHTs^20^. If DHT deployment in clinical research continues to expand without regard for such disparities, we risk perpetuating gaps in the performance of DHTs across groups. Beyond technical performance considerations across groups, DHTs can only lead to more equitable care if they are usable, utile, and accessible to those they purportedly exist to serve. SDoH should be reported in clinical research involving DHTs to foster transparency toward disparities that may be mitigated or perpetuated by the use of such technologies.

There is currently no standardized reporting framework for SDoH in clinical research publications, and SDoH are not systematically documented^21^. However, standardized frameworks for collecting SDoH information in clinical settings have been proposed^22^ and implemented successfully^23^. Similar efforts using standardized assessments with existing medical codes could be implemented in clinical research involving DHTs in order to reveal whether DHTs have been deployed in cohorts representing the spectrum of SDoH in the population. For example, the Logical Observation Identifiers Names and Codes (LOINC®)^24^ is a universal standard for identifying medical observations and contains codes that pertain to SDoH domains.

We evaluated the frequency of reporting on SDoH domains, quantity of unique SDoH domain-groups, and differences in reporting standards among clinical research publications involving DHTs. We also mapped proposed SDoH domains to existing LOINC® codes, which are used in clinical environments to capture SDoH data. By evaluating the current state and proposing a model to capture and facilitate reporting of SDoH data in future clinical trials involving DHTs, we aim to draw attention to the fact that such trials ought to enhance health equity.

## Results

### Frequency of reporting on social determinants of health

**Fig. 1:**
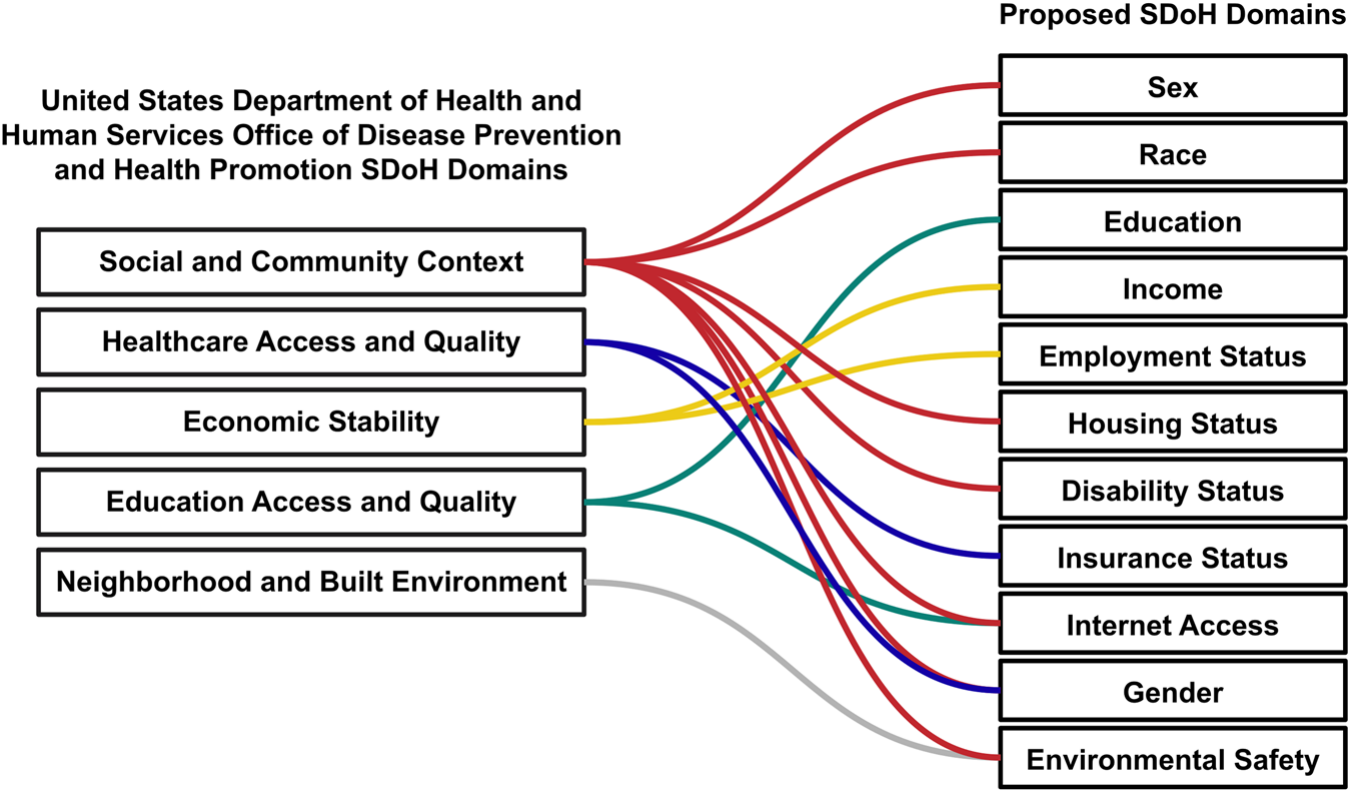
Relationship between ODPHP and proposed SDoH domains. Five SDoH domains established by ODPHP (left) were the basis for the 11 SDoH domains (right) used as the benchmark in this analysis.

We proposed 11 SDoH domains that align to the five SDoH domains established by the United States Department of Health and Human Services Office of Disease Prevention and Health Promotion (ODPHP)^16^ to use as a benchmark for evaluating frequency of reporting SDoH in our analysis (Fig. 1). There were 126 publications in this pilot analysis. Publication dates ranged from 1998 to 2022, though nearly 90% were published in 2020 or later.

Under our proposed 11 domains, there were a total of 1386 potential instances for reporting of SDoH across 126 publications. We identified 207 instances of SDoH domain reporting, accounting for 14.9% of potential instances. Detailed breakdowns of reporting across domains are shown in Table 1. The most frequently reported domain was Sex (94.4% of publications), followed by Race and Education (27.8% and 20.6% of publications, respectively). No other domains were reported in more than 10% of publications. No studies reported on the Gender or Environmental Safety of participants.

**Table 1:** Frequency of SDoH domain reporting within domain-groups.

Social determinant of health domain	Instances reporting (*n*)	Unique domain-groups identified (*n*)
Sex	119	2^a^
Race	35	30
Education	26	46
Income	10	26
Employment status	8	18
Housing status	3	8
Disability status	2	4
Insurance status	2	4
Internet access	2	2
Gender	0	0
Environmental safety	0	0
Total	207^b^	140

^a^In six publications, “Men” and “Women” were interpreted as indicating Male and Female because the publications did not distinguish the use of those terms as indicating Gender rather than Sex, made no mention of Gender, and reported only “Men” and “Women” as the only two domain-groups.

^b^Total number of instances exceeds the 126 total publications because multiple domains could have been reported in a single publication.

### Model framework for capturing social determinants of health data

We identified 45 LOINC® codes addressing our 11 proposed SDoH domains. There were between 1 and 12 codes identified for each domain. We propose a Concept Map (available at: https://github.com/maxgaitan/DigitalHealthTechnologiesSDoH) to demonstrate that existing clinical screening tools can capture SDoH data in clinical trials involving DHTs. Proposed SDoH domains alongside mapped LOINC® codes are also shown in Table 2 for the convenience of the reader.

**Table 2:** Proposed SDoH domains and mapped LOINC® codes.

Proposed SDoH domain	Mapped LOINC® codes (long common name, code)
Sex	Sex, 46098-0
Race	Hispanic, Latino-a, or Spanish origin, 69854-8 Race, 32624-9 Race or ethnicity, 46463-6 Race [PhenX], 56091-2 Race [USSG-FHT], 54134-2 Race or ethnicity OMB.1997, 59362-4 Race or ethnicity [The Position Generator], 67288-1 Race [HHS.ACA Section 4302], 69855-5 Race [AHRQ], 74693-3 Tabulated race [CDC], 80977-2 PhenX—race—ethnic residential segregation protocol 211401, 63038-4
Education	Highest level of education, 82589-3 Highest level of education [US Standard Certificate of Death], 80913-7 Years of education [#]—Reported, 82590-1 What is the highest grade or level of school you have completed or the highest degree you have received [NHANES], 63504-5
Income	How hard is it for you to pay for the very basics like food, housing, medical care, and heating, 76513-1 PhenX—chronic stress protocol 181301, 62942-8 Financial resources [PCAM], 83335-0 Total combined household income range in last year, 77244-2 PhenX—annual family income protocol 011101, 63058-2
Employment status	Current occupational status [SAMHSA], 68505-7 Employment status of family member, 85152-7 Employment status—current, 67875-5 PhenX—environmental exposures—occupation—occupation history protocol 060501, 62522-8
Housing status	Housing status, 71802-3 Alternate residence status [NTDS], 74283-3 Home environment safety and stability [PCAM], 83322-8 Your rent or mortgage is too much [PhenX], 67040-6 PhenX—environmental exposures—plastic exposures at work and home protocol 061401, 62540-0 PhenX—environmental exposures—characteristics of current residence protocol 060101, 62514-5 Household size [#], 86639-2
Disability status	Disability type, 95377-8
Insurance status	Primary insurance, 76437-3 Payment sources, 52556-8 PhenX—health insurance coverage protocol 011501, 63066-5 Health insurance funding was provided, 74186-8
Internet access	Devices used in household to access the internet for learning, 99802-1
Gender	Gender identity, 76691-5
Environmental safety	Home environment safety and stability [PCAM], 83322-8 PhenX—exposures to violence—adult protocol 181401, 62944-4 PhenX—exposures to violence—child protocol 181402, 62945-1 PhenX—neighborhood safety protocol 210901, 63028-5 PhenX—perceived social support—conflict protocol 180701, 62929-5

## Discussion

SDoH domains are infrequently and inconsistently reported in studies utilizing DHTs: only 14.9% of potential instances in our categorization schema were reported. The substantial number of unique domain-groups identified across publications within some domains (e.g., Employment Status) demonstrates these data are not captured in a standardized manner. If study teams are to consider SDoH during planning, recruitment, and reporting of trials, a unified framework under which to do so would enable their efforts. Dozens of existing medical codes in LOINC® alone address our proposed SDoH domains. These codes could be leveraged to capture and subsequently facilitate reporting of SDoH data in future clinical trials involving DHTs.

Under our model of 11 SDoH domains, reporting coverage was variable. Of publications in our sample, most reported on Sex, while a minority reported on Race and Education and less than 10% represented the remaining eight domains. In comparison to randomized controlled trials published in high-impact factor health journals from 2014 to 2020 (i.e., non-DHT-specific literature), reporting in the DHT-specific literature is not more frequent^25^. Our findings demonstrate an opportunity for more data to be reported regarding the cohorts in which DHTs have been deployed in clinical research.

The issue of conflating terms for Gender and Sex^26^ emerged in our analysis. Six studies reported including “men” and “women” but did not distinguish the use of those terms as indicating Gender rather than Sex, made no other mention of Gender, and reported only these two domain-groups. We inferred that these studies were conflating terms for Sex and Gender and recorded them as having reported Sex but not Gender. Analyses of other clinical trials also indicate that reporting on Gender is low, at less than 3%^25,27^. This affirms the need to increase reporting on—and representation of—Gender identities of participants in DHT-enabled clinical research. Reporting of Sex should not be mistaken with representing interests across a spectrum of Gender identity.

Especially in the context of studies involving DHTs, reporting of Internet Access is important because populations without it are affected by health disparities^28^. Only two papers in our analysis reported Internet Access. In both cases, all participants had a computer with Wi-Fi or a smartphone with a data plan. At-home Internet Access can be limited by geographic and socioeconomic factors^29^. Increased reporting on Internet Access in research involving DHTs would help identify domain-groups in need of additional support to participate in clinical trials. This problem also must be addressed through inclusive recruitment inclusion criteria—if Internet Access is required for participation in a given study, those without will continue to be excluded from representation in research. Broadband Internet Access provides an emerging route to healthcare; it is a digital determinant of health that is becoming central to care and threatens to leave behind those without access^30^.

Broadening the lens to other SDoH domains demonstrates a consistent opportunity for increased reporting in the DHT literature. There were 15 instances of reporting across Employment Status, Housing Status, Disability Status, Insurance Status, and Environmental Safety combined, representing less than 3% of potential reporting instances among these domains. When publications reported these domains, data were variably helpful in understanding the potential impact on feasibility of deploying DHTs. For example, among the three publications identified as reporting information on Housing Status, two reported the type of dwelling participants occupied (e.g., one-story house, two-story house, townhouse, etc.). The third captured an indication of housing status (e.g., have housing, worried about losing housing, or do not have housing), data that could be more useful for identifying barriers to participation in DHT research studies. The current variability in reported domain-groups within an SDoH domain highlights that capturing these data using a standardized system (e.g., via LOINC® codes) could enable more meaningful insights into the equitable deployment of DHTs in clinical research.

Our results identify an opportunity for increased inclusivity in the proposed 11 SDoH domains. Participants identified as “Other” in 16 reporting instances, most often with regard to Race. This highlights potential erasure of groups not represented among predetermined options. Restricting participants to a set of options from which to choose to identify is limiting, and many of the publications including an “Other” option report a strictly limited selection of domain-groups (impacted publications may be identified in the Supplemental Table; e.g., “Caucasian, Hispanic/Latino, African American, Asian American, Other”). While our Concept Map proposes tools represented by LOINC® codes to collect data, we recognize that the available responses on these tools are also limited. Increased representation and granularity must be balanced with standardization of participant data across trials. Discretizing domain-groups increases the possibility of certain domain-groups being absorbed into broader categories, but it increases the likelihood of data availability to advocate for greater inclusion for underrepresented domain-groups. For example, we identified 46 domain-groups in the Education domain; the International Standard Classification of Education defines nine Education levels^31^. Standardized stratification of domain-groups would enhance comparability of participant data across trials to inform data-driven recruitment strategies that would reduce gaps in SDoH representation in digital clinical trials.

We acknowledge the importance of intersectionality with regard to the potential impacts and limitations of our perspective. Demographic surveys often allow participants to identify with only one of a given set of domain-groups. Research participants may self-identify with multiple domain-groups; allowing selection of only one SDoH domain-group in survey responses may lead to erasure of those unassigned. Some domains contain mutually exclusive domain-groups, such as Internet Access and Income. Others, however, such as Race and Gender, could allow participants to identify with multiple domain-groups. Allowing multiple selection on such surveys and reporting intersections in DHT-enabled clinical research publications would preserve discrete grouping for data reporting and aggregation while empowering intersectionality for participants.

Ability to write and/or speak in the primary language of the respective geographic region of each study was not proposed as one of the 11 SDoH domains derived from the ODPHP SDoH domain objectives. We noted some studies in our sample excluded participants who were unable to understand written or spoken English; such exclusion criteria introduce sampling bias. Language proficiency is known to impact clinical care and health outcomes^32^, and trials involving DHTs should strive to be inclusive of non-English-speaking participants^33^. If our proposed framework were expanded beyond the scope from ODPHP, there are numerous instruments with LOINC codes (e.g., 66574-5, 54899-0, 45402-5) that could be used to capture information on language proficiency.

Recognizing that study sample size may impact reporting on SDoH, where smaller pilot studies might report fewer metrics or less frequently than larger trials, we explored differences between publications based on sample size. Of the 126 publications in our analysis, 69 had samples smaller than 50, representing 759 potential reporting instances in our framework. There were 99 total reports in this sub-sample, or 13.0% of potential instances. Fifty-eight publications had samples greater than or equal to 50, representing 638 potential SDoH reporting instances. We observed 103 total reports of SDoH in this sub-sample, or 16.1% of potential instances. This exploration of the data reveals that the smaller studies may diminish the average reporting metrics slightly, but are not uniquely responsible for the infrequent reporting of SDoH in studies utilizing DHTs.

Our perspective is subject to limitations inherent to our sample of publications. Due to our method of sampling (see “Methods”), the publications in this analysis have a recency bias—the majority of publications included in this analysis were published between 2020 and 2022—meaning our findings may not generalize to earlier years. However, the FDA has released guidance^34,35^ in recent years on enhancing reporting of diversity metrics. In fact, an analysis of pediatric clinical trials showed increasing reporting of Race and Ethnicity in clinical trials from 2011 to 2020^27^. Given growing attention to SDoH and the fact that our analysis was weighted toward recent evidence, our results may reflect even greater reporting than would have been found in previous years and decades. Additionally, our analysis is representative only of published peer-reviewed articles; publication bias suggests this sample is less representative of diverse participant cohorts^36^ and of studies without positive results^37^. The latter could be particularly important to understand if deploying DHTs does not support successful outcomes among diverse cohorts in clinical research. Finally, with regard to our Concept Map, we acknowledge that within a given SDoH domain the proposed LOINCs are not mutually exclusive or collectively exhaustive. Using all of them within a domain would not necessarily be useful, nor would doing so address the full breadth of the domain.

Our findings reveal the need for increased reporting of SDoH data in clinical research that deploy DHTs. The CONSORT-Equity framework for reporting on health equity currently serves as the accepted standard for randomized trials^38^. However, it is targeted toward trials reporting on health equity; similar guidelines for research publications involving DHTs would lend transparency to the study cohorts in DHT-enabled trials and could motivate more intentional inclusivity in future trials. A starting point for capturing the relevant data exists in medical coding systems, as highlighted in our Concept Map for LOINC® codes relevant to proposed SDoH domains. The infrastructure for establishing a framework for reporting on SDoH will require input from key stakeholders, including participants, researchers, and subject-matter experts. Standardized SDoH variables resulting from those groups’ input should be included in the research reporting process. Federal funding agencies ought to mandate inclusion and collection of these variables as criteria for funding eligibility. In fact, models for this proposed requirement already exist. The National Institutes of Health (NIH) requires that proposed research projects address inclusivity on the basis of sex, gender, race, ethnicity, and age^39^, and the Food and Drug Administration is developing requirements for representation of racial and ethnic populations in clinical trials^40^. Additionally, reporting of SDoH variables should be strongly recommended by academic journals as criteria for publication. Without data on SDoH in clinical research, equitable digital innovation will be stifled for historically underrepresented groups.

## Methods

### Evidence sourcing

The HumanFirst Atlas platform (https://app.gohumanfirst.com/atlas) catalogs DHTs and supporting evidence in order to support decentralized trials and distributed care. Evidence types include peer-reviewed research publications, clinical trials, US Food and Drug Administration (FDA) documentation, conference abstracts, and industry white papers. Evidence is considered in-scope for Atlas if it reports utilization of at least one DHT to capture digital health measures. Evidence is added daily using a proprietary method involving automated and manual identification of relevant literature from publicly available sources. Our evidence sourcing spans across all years, but is weighted toward the most recent 3-5 years because of our intent to catalog the most up-to-date evidence in Atlas. We used a purposive sampling method from our existing evidence for this analysis, in which peer-reviewed research publications were included sequentially as they appeared in our queue for inclusion into Atlas, with no further targeting. Because our aim was to evaluate reporting of SDoH domains in peer-reviewed publications, evidence appearing sequentially from clinical trial records, industry white papers, FDA documentation, and conference abstracts was excluded. The analysis was carried out between July 8, 2022 and August 29, 2022 and resulted in a sample size of 126 publications. Because our data were collected from published literature, the activities did not constitute human subjects research and neither IRB approval nor notice of exemption were deemed required.

### Social determinants of health domains

Social determinants can impact health. To better understand SDoH factors at a systems level, broader determinants are sometimes referred to as SDoH domains. For example, SDoH factors such as public school system quality and parental educational status, fall into the SDoH domain of Education. To establish a set of SDoH domains on which to benchmark the publications in our analysis, we reviewed the five SDoH domains established by the ODPHP^16^. These are Social and Community Context, Health Care Access and Quality, Economic Stability, Education Access and Quality, and Neighborhood and Built Environment. We expanded on these domains to form a list of 11 SDoH domains that would address the broader ODPHP domains if captured in clinical research data. These are Sex, Race, Education, Income, Employment Status, Housing Status, Internet Access, Disability Status, Insurance Status, Gender, and Environmental Safety. These were derived by reviewing each of the Related Objectives that are categorized under each of the five ODPHP domains. While not all objectives could be accounted for across published health literature (e.g. the Related Objective ‘Increase the proportion of the voting-age citizens who vote—SDOH-07’), those that were more closely related to health outcomes were used as a basis for the expanded 11 domains (e.g. ‘Increase the proportion of people with health insurance—AHS-01’)^16^. The 126 publications were evaluated for reports of information about participants that addressed these 11 domains.

### Data extraction

We first documented whether (“Not Reported” or “Reported”) each of the 11 individual SDoH domains was represented in each of the 126 publications. To be documented as “Reported”, a domain must have been described as representing at least one participant in the study in the Methods or Results sections. We also documented the underlying domain-group(s) and associated sample size(s) representing each domain. For example, in a publication reporting employment, domain-groups might include (but not be limited to) “Full time, Part time, Retired, Homemaker, Medical leave, or Unemployed,” each with an associated sample size. The full dataset, including the domain-groups and associated sample sizes, is available in the Supplemental Table.

### Concept Map

The LOINC® coding system is an international standard for identifying health measurements, observations, and documents. The terms for our 11 proposed SDoH domains were searched in the LOINC® database^24^ to identify 45 codes that represent concepts captured by the 11 domains. For an SDoH domain that maps to multiple LOINC® codes, not all codes need to be implemented to adequately map the SDoH domain (e.g., Race maps to 11 distinct LOINC® codes, and implementing only one code would suffice). The relationships between our proposed domains and existing medical codes were coded in a Health Level Seven International (HL7®) Fast Healthcare Interoperability Resources (FHIR®) Concept Map (https://hl7.org/fhir/conceptmap.html) in Java Script Object Notation (JSON) format (https://github.com/maxgaitan/DigitalHealthTechnologiesSDoH).

### Statistical analysis

Instances of reporting of each SDoH domain were summarized across publications. Unique domain-groups within each domain across all publications were also summarized. All data analysis was conducted using Google Sheets spreadsheet software (Google LLC).

## Data Availability

To access the proposed Concept Map with LOINC® codes, please visit: https://github.com/maxgaitan/DigitalHealthTechnologiesSDoH. To access the Supplemental Table containing the studies analyzed in this report, please visit https://osf.io/v3hb9/.

## Code availability

The underlying code for this study is available in the Digital Health Technologies SDoH GitHub repository and can be accessed via this link: https://github.com/maxgaitan/DigitalHealthTechnologiesSDoH.
